# Cancer Detection Rates of Systematic and Targeted Prostate Biopsies after Biparametric MRI

**DOI:** 10.1155/2020/4626781

**Published:** 2020-04-03

**Authors:** Maudy C. W. Gayet, Anouk A. M. A. van der Aa, Harrie P. Beerlage, Bart Ph Schrier, Maaike Gielens, Roel Heesakkers, Gerrit J. Jager, Peter F. A. Mulders, Hessel Wijkstra

**Affiliations:** ^1^Department of Urology, Jeroen Bosch Hospital, Henri Dunantstraat 1, 5223 GZ, ‘s-Hertogenbosch, Netherlands; ^2^Department of Electrical Engineering, Eindhoven University of Technology, De Rondom 70, 5612 AP, Eindhoven, Netherlands; ^3^Department of Urology, AMC University Hospital, Meibergdreef 9, 1105 AZ, Amsterdam, Netherlands; ^4^Department of Radiology, Jeroen Bosch Hospital, Henri Dunantstraat 1, 5223 GZ, ‘s-Hertogenbosch, Netherlands; ^5^Department of Urology, Radboudumc University Hospital, Geert Grooteplein Zuid 10, 6525 GA, Nijmegen, Netherlands

## Abstract

**Objective:**

To compare prostate cancer detection rates (CDRs) and pathology results with targeted prostate biopsy (TB) and systematic prostate biopsy (SB) in biopsy-naive men.

**Methods:**

An in-patient control study of 82 men undergoing SB and subsequent TB in case of positive prostate MRI between 2015 and 2017 in the Jeroen Bosch Hospital, the Netherlands.

**Results:**

Prostate cancer (PCa) was detected in 54.9% with 70.7% agreement between TB and SB. Significant PCa (Gleason score ≥7) was detected in 24.4%. The CDR with TB and SB was 35.4% and 48.8%, respectively (*p*=0.052). The CDR of significant prostate cancer with TB and SB was both 20.7%. Clinically significant pathology upgrading occurred in 7.3% by adding TB to SB and 22.0% by adding SB to TB.

**Conclusions:**

There is no statistically significant difference between CDRs of SB and TB. Both SB and TB miss significant PCas. Moreover, pathology upgrading occurred more often by adding SB to TB than vice versa. This indicates that the omission of SB in this study population might not be justified.

## 1. Introduction

Because of the well-known limitations of systematic prostate biopsies (SB), there is an increasing focus on different imaging techniques in the diagnosis of prostate cancer (PCa) for the last decades [1, 2]. Merging anatomic and functional information using multiparametric magnetic resonance imaging (mpMRI), consisting of T2-weighted imaging, diffusion weighted imaging (DWI), and dynamic contrast enhanced (DCE) imaging, resulted in improved accuracy for detection of PCa compared to T2 images alone [3–5]. Standardized mpMRI interpretation and reporting was proposed by the developers of PI-RADS and is used internationally. Recently, the PI-RADS v2 was published [6]. Approaches of taking magnetic resonance- (MR-) guided biopsies of the prostate include direct “in-bore” MR-guided biopsies, cognitive fusion, and MR/ultrasound (MR/US) software-based image fusion techniques. In “in-bore” MR-guided techniques, biopsies are taken during real-time MRI. Concerning the two other techniques, magnetic resonance imaging (MRI) is performed before biopsy, and biopsies are taken using cognitive or software-based MR/ultrasound (US) image fusion techniques.

To date, there is an ongoing debate whether adding DCE to T2-weighted imaging and DWI is of additional value. DCE seems to be of limited value, and biparametric MRI (bpMRI) is considered to be a faster and cheaper alternative to mpMRI [7, 8]. Cancer detection rates (CDRs) of MR TB and SB were compared in a systematic review and meta-analysis by Schoots et al. [9]. Men, either biopsy-naive or after prior negative biopsy, with a clinical suspicion of PCa and a subsequent positive mpMRI were included in the analysis. Comparing MR TB with SB resulted in equal CDRs.

Regarding clinically significant PCa only, a higher CDR was found for MR TB. Moreover, less clinically insignificant PCas were found using MR TB. These are promising results; however, long term follow-up of negative mpMRIs or negative TB is not available. Besides, most studies are performed in large tertiary centers with a mixed population of biopsy-naive men and men after prior negative biopsy. It would therefore be premature to omit SB. Additional prospective trials are urgently needed, especially in lower volume centers [10, 11].

For this reason, the aim of this study was comparing CDRs and pathology results of PCa in MR/US fusion TB and SB in biopsy-naive men in a secondary health-care centre, using an in-patient study design in which SB is followed by TB.

## 2. Materials and Methods

### 2.1. Study Population

Between January 2016 and May 2017, 90 biopsy-naive men were included in this prospective in-patient control study. Subjects aged 50–75 years, had a clinical suspicion of PCa due to an elevated PSA (4–30 *μ*g/L for men aged 50–65 years and 10–30 *μ*g/L for men aged 66–75 years). Men with a history of PCa or contraindications for MRI were excluded. Patients were informed about study procedures, risks, and benefits and were included after written informed consent had been obtained. Ethical approval was granted by “Medical Ethical Committee Brabant.” The trial was registered in The Netherlands National Trial Register with reference NTR5787.

### 2.2. Protocol

All bpMRI scans were performed in the Jeroen Bosch hospital before biopsy using a 3T MR scanner (Magnetom Verio, Siemens) and a 6-channel body-coil for signal reception. MRI consists of anatomical T2-weighted imaging coupled with diffusion-weighted images. Two of three experienced MRI radiologists (one radiologist with one year experience and residency in abdominal radiology and two abdominal radiologists with 8 and 25 years of experience, respectively) independently denoted regions of interest (ROI). Consensus was achieved in case deviations occurred between the ROI determinations. In case of a positive MRI (PI-RADS ≥3), MR images were loaded into the Navigo™ system prior to the biopsy procedure. Then, on the axial T2 images, prostate outlines and the outlines of ROI were marked by one of the clinicians (MGa) in consultation with one of the radiologists (MGi).

A clinician with 5 to 10 years experience performed the prostate biopsy sessions. After administration of prophylactic antibiotics (oral ciprofloxacin 500 mg) and with the patient in the lateral decubitus position, a BK medical ultrasound machine (type 2202), the Navigo™ system, and a sidefire BK medical probe (type 8808, 6–10 MHz) were used to image the prostate transrectally. After volume measurement and contouring using the Navigo™ system, a periprostatic block was given. The SB protocol consisted of transrectal biopsies in the lateral zones of the base, midzone, and apex. In total, 12 cores per biopsy procedure were taken using a Bard magnum gun with an 18-gauge needle and 22 mm cores. In case of a hypoechoic lesion, an extra core was allowed. The clinician taking the SB was blinded for the mpMRI results. Prostate cores were submitted to the pathology laboratory using the Smart-BX™ device (UC-Care, Israel), which is a preservation technology, allowing supplying cores in a stretched form on a cassette. Core specimens were divided in two containers (left: 6 cores and right: 6 cores). During the biopsy procedure, the first core from each zone was inked for identification.

After the SB protocol, the clinician was unblinded for the MR images. A maximum of 4 ROIs were marked in these images by the radiologist. When ROIs were marked in the Navigo™ fusion system, additional TB cores were taken, with a minimum of one core per ROI, with a maximum of 4 cores in total. Core specimens were sent to the pathology laboratory in separate cassettes. Two definitions of clinically significant PCa were used in our analysis. The first one was defined as Gleason score ≥7. The second one was defined as the Gleason score ≥7 or ≥3 cores with a Gleason score 6.

### 2.3. Statistics

Demographic features and baseline characteristics are summarized for all participating patients. The McNemar test was used to compare the prevalences of PCa obtained with each of the individual methods (SB versus TB). To assess whether TB results in a higher Gleason score compared to SB, the percentage with 95% confidence interval is presented. A *p* value of less than 0.05 is considered statistical significant. All analyses were performed with IBM Statistics Version 22.

## 3. Results

### 3.1. Patient Demographics

**Figure 1:**
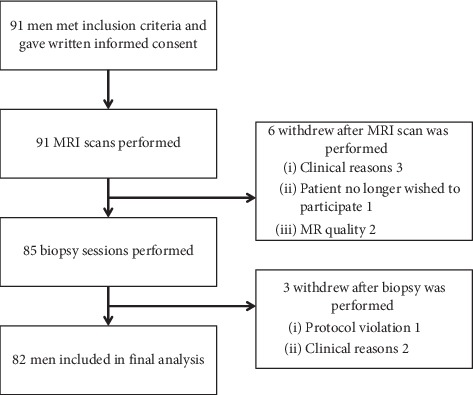
Flow chart.

During the study period, 91 men were included. After the exclusion of 9 men, 82 (90.1%) men were included in the final analysis; 67 (81.7%) in group 1 (age 50–65, PSA 4–30 *μ*g/L) and 15 (18.3%) in group 2 (age 66–75, PSA 10–30 *μ*g/L). A flow chart of the study is shown in Figure 1. The mean age was 62.0 years, the mean PSA level was 6.6 *μ*g/L, and mean prostate volume was 44.5 cm^3^. In 20 subjects (24%), a hypoechoic lesion was found on TRUS. In all cases, this lesion was sampled with one or two cores. Overall, a mean of 12.1 biopsy cores was taken during SB. In 59 subjects (72%), at least one ROI (PI-RADS ≥3) was found on MRI, with a mean of 1.1 lesions per subject. Sixteen subjects (20%) had an anterior lesion on MRI. 28% of the subjects without a ROI on MRI underwent SB only. For all subjects together, a mean of 2.2 targeted biopsy cores was taken. For subjects with a ROI on MRI that underwent TB, a mean of 3.0 biopsy cores was taken. Patient demographics for all patients and both subgroups are listed in Table 1.

### 3.2. Comparison of TB and SB

Tables 2 and 3 present a summary of biopsy findings.  Table 2 shows a comparison between pathology outcomes of TB and SB using the first definition of csPCa.  Table 3 shows the same comparison using the second definition of csPCa. Overall, PCa was detected in 54.9%, with 70.7% agreement between TB and SB. Significant PCa (Gleason score ≥7) was detected in 24.4%. The CDR with TB and SB only was 35.4% and 48.8%, respectively (*p*=0.052).

The CDR of significant prostate cancer for both TB and SB was 20.7% (*p*=1.000). In patients with a negative MRI (*n* = 24), CDRs were 37.5% and 0.0% for all PCas and significant PCa only, respectively.

The orange zone in Table 2 denotes patients in whom there was an added clinical value of SB above TB, due to an upgrade from “no cancer” to “cancer” or from “insignificant cancer” to “significant cancer.” The blue zone indicates patients in whom an added value of clinical importance of TB above SB was found. Clinically significant pathology upgrading took place in 7.3% by TB to SB, designated by the blue zone, and 22.0% by adding SB to TB, designated by the orange zone. Significant PCa was found with SB and not with TB in 3/17 (17.6%) of the cases. Significant PCa was found with TB and not with SB in 3/17 (17.6%) of the cases.

Supplementary Table S1 shows an additional comparison of Gleason scores between TB and SB. 28.0% of subjects underwent SB only because of a negative MRI. Within this group, the CDR for all PCa and significant PCa was 37.5% and 0.0%. In patients with an anterior lesion (*n* = 16), the CDR for all PCa and significant PCa was 87.5% and 43.8%.

A subanalysis was performed in group 1 (age 50–65, PSA 4–30 *μ*g/L) and group 2 (age 66–75, PSA 10–30 *μ*g/L). Supplementary Tables S2 and S3 show pathology outcomes for both group 1 and 2. Regarding group 1, CDRs for SB and TB were 43.3% and 31.1%, respectively. CDRs of significant PCas for SB and TB were 19.4% and 17.9%. Regarding group 2, CDRs for SB and TB were 73.3% and 53.3%. CDRs of significant PCas for SB and TB were 26.7% and 33.3%, respectively.

### 3.3. Per Lesion Analysis

In 82 patients, a total of 92 lesions were denoted on mpMRI. Twenty-seven patients had 1 lesion, 28 patients had 2 lesions, and 3 patients had 3 lesions on mpMRI.  Table 4 shows detection rates for clinically significant prostate cancer for both definitions per PI-RADS classification score.

## 4. Discussion

We evaluated CDRs and pathology outcomes of TB versus SB in a secondary health-care centre. No significant differences between CDRs were found. However, both SB and TB missed significant PCas depending of the definition used. In subjects with a negative MRI, no ≥Gleason 7 PCas were found.

In 2015, Schoots et al. performed a meta-analysis and concluded that, in men with a clinical suspicion of PCa and a positive MRI, TB and SB did not differ in overall PCa detection [9]. In a subgroup analysis of only biopsy-naive men, a similar detection of overall PCa for TB and SB was found. In men after prior negative biopsy, CDRs of 37% and 24% were found. Furthermore, TB showed a lower detection of insignificant PCa compared to SB. In contrast to Schoots et al., in our study, a CDR with TB of 35.4% was found. There are several factors that may explain this difference. First of all, and probably the most important one, the majority of studies in the meta-analysis included patients with a positive MRI only. With a negative MRI, the chance of finding (significant) PCa is most probably lower, and therefore, in our study, this could contribute to the lower total CDR found. Secondly, patients in the meta-analysis were characterized by a higher mean PSA, which also increases the chance of finding PCa. Our study design is not comparable to the one of the PROMIS trial, but a similar percentage of positive MRIs was found, which suggests that the inferior CDR with TB in our study is not caused by allocating too many lesions on MRI [12].

The present study has several strengths and weaknesses. Strengths are its in-patient design, the contouring of the MR images by a radiologist and a clinician together in preparation of the MR/US fusion, and the double reader MRI protocol. Furthermore, in assessing the clinical value of mpMRI, men with a negative MRI should not be disregarded and were, therefore, included in our analysis. As in all comparable studies, one of the main limitations of our study is the suboptimal reference test based on SB. This is a well-known limitation in all studies concerning the diagnosis of PCa based on biopsy results only. In the recently presented PROMIS study, template prostate mapping (TPM) was used as the reference test [12]. TPM has an area under the curve of 90%. It is, therefore, superior compared to TRUS or any other biopsy technique [13, 14]. Taking the burden of general or epidural anaesthesia and transperineal biopsy into account, this reference test should, therefore, be considered when comparing diagnostics of PCa. Another well-known limitation and discussion of targeted biopsy studies is the definition for significant PCa. There are several definitions for significant PCa based on the pathology results of SB. However, TB is very different from SB, both in its number and its origin. By means of increasing experience and research, a new definition should be developed. Because of lack of a commonly used definition for significant PCa on TB, one of the definitions used in this study are the well-known Epstein criteria [15]. Because more cores were taken using SB compared to TB, the endpoint of the study is inherently biased towards SB. Therefore, analyses were also performed using Gleason ≥7 as a cutoff for clinical significance.

In 12 cases, pathology results of TB showed a Gleason 3 + 3 = 6 disease, of which 2 with more than 2 cores which, therefore, were identified as clinically significant when using the Epstein criteria [15]. The MR/US fusion system used is Navigo™, which is not widely used and specific studies reporting the accuracy are still lacking. A recent study of Westhoff et al. showed a median distance to the lesion centre of 3.15 mm using a fusion system that uses rigid fusion and transrectal prostate biopsy [16]. One of the main problems in MR/US TB is that it is difficult to verify the exact location of the needle. Therefore, a negative targeted core can also be due to a location error. However, also in negative MRIs with omission of TB, in 9%, significant tumours are found [9]. In the latter, a location error is obviously not the cause. Finally, our study design, in which TB is performed after 12-core SB, might have played a role in the results. It is assumed that prostate biopsy results in swelling of the prostate gland. This volume change might lead to diminished accuracy of TB.

Another well-known limitation in PCa diagnosis is the absence of a clear PSA cutoff value for prostate biopsy [17]. Because of the lack of a clear guideline, this study contains two different age-PSA groups based on the fact that age is independently associated with PSA level [18]. Both in the PROMIS and PRECISION trial, a mpMRI-first workflow with or without TB is proposed because of its noninferiority compared to the regular TRUS workflow [12, 19]. However, it is unknown how many clinically significant PCas are missed using such a workflow compared to mpMRI followed by SB. In the PROMIS study, the mpMRI-first workflow missed 9.3% significant PCas (defined as Gleason score ≥4 + 3) using TPM as a reference test. In our study, we found 12.5% significant PCas (defined as Gleason score ≥3 + 4) on SB. mpMRI with or without TB might be superior to SB, but the omission of SB should be considered carefully. Moreover, recent studies about the cost-effectiveness of mpMRI show a clear benefit for mpMRI-first strategies compared to TRUS-biopsy-first strategies [20, 21]. Concerning the results, omission of SB in the initial diagnosis of PCa in our study population might not be justified. Additional clinical trials using a study design in which a mpMRI-only workflow is compared to a mpMRI-SB combination workflow are needed. Because of the current lack of reliable diagnostic modalities, the next step to further improve prostate cancer diagnosis will be finding the most accurate and cost-effective combination of various modalities.

## 5. Conclusions

In our study population, CDRs using TB are similar to CDRs of SB. Furthermore, pathology upgrading occurred more often by adding SB to TB than vice versa. This indicates that the omission of SB in this study population might not be justified, and a combination of TB and SB is, therefore, recommended.

## Figures and Tables

**Table 1:** Patient demographics.

Variable	All
No. of men	82
Age, mean (SD), *y*	62.0 (5.2)
PSA, median (IQR), *μ*g/L	6.6 (5.6–10.1)
Prostate volume, median (IQR), cm^3^	44.5 (31.8–59.3)
Men with lesion(s) on mpMRI, no. (%)	58 (71)
Number of lesions on mpMRI, mean (SD)	1.1 (0.9)
Patients with anterior lesions, no. (%)	16 (20)
Index lesion score on mpMRI^a^, no. (%)	
PI-RADS 3	23 (28)
PI-RADS 4	14 (17)
PI-RADS 5	22 (27)
Tumor staging^b^	
<T2	56 (68)
T2	15 (18)
T3	11 (13)
T4	0 (0)
Systematic biopsy cores per patient, mean (SD)	12.1 (0.4)
Targeted biopsy cores per patient, mean (SD)	2.2 (1.6)

^a^Highest PI-RADS score. ^b^Clinical staging based on digital rectal examination.

**Table 2:** Whole group pathology outcomes of systematic biopsy and targeted biopsy.

Targeted biopsy	Systematic biopsy			Total
	No cancer	Gleason 6	≥Gleason 7	
Not performed	15	**9**	**0**	24
No cancer	22	**6**	**1**	29
Gleason 6	*3*	7	**2**	12
≥Gleason 7	*2*	*1*	14	17
Total	42	23	17	82

Pathology outcomes per patient for systematic biopsy and targeted MR/US fusion biopsy. The numbers in bold indicate patients with a pathology upgrade of clinical importance with systematic biopsy. The numbers in italics indicate patients with a pathology upgrade of clinical importance with targeted biopsy.

**Table 3:** Whole group pathology outcomes of systematic biopsy and targeted biopsy.

Targeted biopsy	Systematic biopsy			Total
	No cancer	≤2 cores Gleason 6	≥3 cores Gleason 6 or ≥Gleason 7	
Not performed	15	**6**	**3**	24
No cancer	22	**3**	**4**	29
≤2 cores Gleason 6	*2*	4	**4**	10
≥3 cores Gleason 6 or ≥Gleason 7	*3*	0	16	19
Total	42	13	27	82

Pathology outcomes per patient for systematic biopsy and targeted MR/US fusion biopsy. The numbers in bold indicate patients with a pathology upgrade of clinical importance with systematic biopsy. The numbers in italics indicate patients with a pathology upgrade of clinical importance with targeted biopsy.

**Table 4:** Biopsy yield based on PI-RADS stratitication.

Index lesion	Results targeted biopsy only			Results total biopsy			Total
	No cancer	≥3 cores Gleason 6 or ≥gleason 7	≥Gleason 7	No cancer	≥3 cores Gleason 6 or ≥Gleason 7	≥Gleason 7	
*PI-RADS 3*							
*n*	21	0	0	18	3	2	23
%	91.3%	0.0%	0.0%	78.3%	13.0%	8.7%	

*PI-RADS 4*							
*n*	5	5	5	4	8	6	14
%	35.7%	35.7%	35.7%	28.6%	57.1%	42.9%	

*PI-RADS 5*							
*n*	4	14	12	1	15	13	22
%	18.2%	63.6%	54.6%	4.5%	68.2%	59.1%	

## Data Availability

The clinical data used to support the findings of this study are available from the corresponding author upon request.

## Supplementary Materials

Supplementary Materials Supplementary table 1: whole group Gleason scores of systematic biopsy and targeted biopsy. Supplementary table 2: pathology outcomes of systematic biopsy and targeted biopsy in group 1. Supplementary table 3: pathology outcomes of systematic biopsy and targeted biopsy in group 2 Re.
