# Supplementary material for: Cancer Detection Rates of Systematic and Targeted Prostate Biopsies after Biparametric MRI
Source: Prostate Cancer. 2020 Apr 3;2020:4626781. doi: 10.1155/2020/4626781 (PMC7157788; doi:10.1155/2020/4626781)
Supplement: Supplementary Materials — Supplementary table 1: whole group Gleason scores of systematic biopsy and targeted biopsy. Supplementary table 2: pathology outcomes of systematic biopsy and targeted biopsy in group 1. Supplementary table 3: pathology outcomes of systematic biopsy and targeted biopsy in group 2 Re. [file 4626781.f1.docx]

## Supplementary table 1: Whole group Gleason scores of systematic biopsy and targeted biopsy

| Targeted biopsy | Systematic biopsy | | | | | | | Total | |
| --- | --- | --- | --- | --- | --- | --- | --- | --- | --- |
|  | No cancer | Gleason 3+3 | Gleason 3+4 | Gleason 4+3 | Gleason 8 | > Gleason 8 |  | |  |
| Not performed | 15 | 9 | 0 | 0 | 0 | 0 | 24 | |  |
| No cancer | 22 | 6 | 1 | 0 | 0 | 0 | 29 | |  |
| Gleason 3+3 | 3 | 7 | 1 | 1 | 0 | 0 | 12 | |  |
| Gleason 3+4 | 0 | 0 | 5 | 2 | 0 | 0 | 7 | |  |
| Gleason 4+3 | 1 | 0 | 1 | 3 | 0 | 0 | 5 | |  |
| Gleason 8 | 1 | 1 | 0 | 0 | 2 | 0 | 4 | |  |
| > Gleason 8 | 0 | 0 | 0 | 0 | 0 | 1 | 1 | |  |
| Total | 42 | 23 | 8 | 6 | 2 | 1 | 82 | |  |

Pathology outcomes per patient for systematic biopsy and targeted MR/US fusion biopsy.

The orange zone indicates patients with a pathology upgrade with systematic biopsy.

The blue zone indicates patients with a pathology upgrade with targeted biopsy.

## Supplementary table 2: Pathology outcomes of systematic biopsy and targeted biopsy in group 1

| Targeted biopsy | Systematic biopsy | | | Total |
| --- | --- | --- | --- | --- |
|  | No cancer | Gleason 6 | Gleason ≥ 7 |  |
| Not performed | 15 | 5 | 0 | 20 |
| No cancer | 21 | 4 | 1 | 26 |
| Gleason 6 | 1 | 6 | 2 | 9 |
| Gleason ≥ 7 | 1 | 1 | 10 | 12 |
| Total | 38 | 16 | 13 | 67 |

Pathology outcomes per patient for systematic biopsy and targeted MR/US fusion biopsy.

The orange zone indicates patients with a pathology upgrade of clinical importance with systematic biopsy.

The blue zone indicates patients with a pathology upgrade of clinical importance with targeted biopsy.

## Supplementary table 3: Pathology outcomes of systematic biopsy and targeted biopsy in group 2

| Targeted biopsy | Systematic biopsy | | | Total |
| --- | --- | --- | --- | --- |
|  | No cancer | Gleason 6 | Gleason ≥ 7 |  |
| Not performed | 0 | 4 | 0 | 4 |
| No cancer | 1 | 2 | 0 | 3 |
| Gleason 6 | 2 | 1 | 0 | 3 |
| Gleason ≥ 7 | 1 | 0 | 4 | 5 |
| Total | 4 | 7 | 4 | 15 |

Pathology outcomes per patient for systematic biopsy and targeted MR/US fusion biopsy.

The orange zone indicates patients with a pathology upgrade of clinical importance with systematic biopsy.

The blue zone indicates patients with a pathology upgrade of clinical importance with targeted biopsy.
